# A Polysomnographic Study of Parkinson's Disease Sleep Architecture

**DOI:** 10.1155/2015/570375

**Published:** 2015-10-04

**Authors:** Daniel Martinez-Ramirez, Sol De Jesus, Roger Walz, Amin Cervantes-Arriaga, Zhongxing Peng-Chen, Michael S. Okun, Vanessa Alatriste-Booth, Mayela Rodríguez-Violante

**Affiliations:** ^1^Department of Neurology, University of Florida Center for Movement Disorders and Neurorestoration, Gainesville, FL 32607, USA; ^2^Departamento de Clínica Médica, Centro de Neurociências Aplicadas, HU, Universidade Federal de Santa Catarina, 88040-970 Florianópolis, SC, Brazil; ^3^Laboratorio Clínico de Enfermedades Neurodegenerativas, Clinica de Trastornos del Movimiento, Instituto Nacional de Neurología y Neurocirugía, 14269 Ciudad de México, DF, Mexico; ^4^Department of Neurosurgery, University of Florida Center for Movement Disorders and Neurorestoration, Gainesville, FL 32610, USA; ^5^Unidad de Medicina del Sueño, Instituto Nacional de Neurología y Neurocirugía, 14269 Ciudad de México, DF, Mexico

## Abstract

Sleep disturbance is a common nonmotor phenomenon in Parkinson's disease (PD) affecting patient's quality of life. In this study, we examined the association between clinical characteristics with sleep disorders and sleep architecture patterns in a PD cohort. Patients underwent a standardized polysomnography study (PSG) in their “on medication” state. We observed that male gender and disease duration were independently associated with obstructive sleep apnea (OSA). Only lower levodopa equivalent dose (LED) was associated with periodic limb movement disorders (PLMD). REM sleep behavior disorder (RBD) was more common among older patients, with higher MDS-UPDRS III scores, and LED. None of the investigated variables were associated with the awakenings/arousals (A/A). Sleep efficiency was predicted by amantadine usage and age, while sleep stage 1 was predicted by dopamine agonists and Hoehn & Yahr severity. The use of MAO-B inhibitors and MDS-UPDRS part III were predictors of sleep stages 2 and 3. Age was the only predictor of REM sleep stage and gender for total sleep time. We conclude that sleep disorders and architecture are poorly predictable by clinical PD characteristics and other disease related factors must also be contributing to these sleep disturbances.

## 1. Introduction

Sleep disturbance is a common nonmotor phenomenon in Parkinson's disease (PD) patients with a prevalence reported to vary from 40% to 90% [1]. Sleep patterns and architecture change over time in the aging brain and are thought to decrease in both quality and quantity in the elderly population. The neurodegenerative disease process involves multiple motor and nonmotor networks thought to ultimately contribute to the degeneration of normal sleep cycles beyond that seen in aging alone. The potential result of this degeneration is an increased severity in sleep dysfunction and a heterogeneity in sleep disturbance clinical presentations in the PD patient [1, 2]. Complaints of sleep dysfunction often predate motor symptoms in PD and can include rapid eye movement behavior sleep disorder, excessive daytime somnolence, obstructive sleep apnea, impaired initiation and maintenance of sleep, and restless legs and also can include other parasomnias [3]. Proper vigilance is necessary as the coexistence of disrupted sleep in PD affects quality of life, motor symptoms, cognitive function, and caregiver burden.

Sleep disturbance in the PD population has traditionally been considered to be multifactorial. Contributing factors identified have included the nature of the neurodegenerative brain changes, aging, dopaminergic medications, disease severity, and comorbid neuropsychiatric disorders [4–6]. However, most of the studies have used subjective sleep measurement. In the present study, we aimed to determine the associations between clinical characteristics with polysomnography (PSG) diagnosed sleep disorders and sleep architecture patterns in a PD cohort.

## 2. Materials and Methods

The National Institute of Neurology and Neurosurgery (NINN) Institutional Review Board and Ethics Committee approved the study. Written informed consent was provided from study participants for research purposes.

### 2.1. Study Design

The present study was a cross-sectional and descriptive data/chart review study conducted at the NINN-Movement Disorders Clinic in Mexico City. Data was collected during the period of June 2009 to May 2013.

### 2.2. Study Participants

Subjects with clinical diagnosis of PD made by a movement disorders fellowship-trained specialist, who fulfilled the UK Parkinson's Disease Society Brain Bank Clinical Diagnostic Criteria [7], were eligible for inclusion. PD patients who had a PSG study performed within a year for sleep complaints and with a complete data/chart were included in the final analysis.

### 2.3. Data Sources and Measurements

The following variables of interest were extracted reviewing each subject's chart. Demographics, clinical characteristics, and the Movement Disorders Society-Unified Parkinson Disease Rating Scale (MDS-UPDRS) were extracted from each subject's chart [8]. We also documented the presence of motor fluctuations or levodopa-induced dyskinesias (LID); dopaminergic medications such as levodopa (LD), dopamine agonists (DA), levodopa equivalent dosage (LED), and if on monotherapy or polytherapy of dopaminergics; and other medications, such as MAO-B inhibitors, amantadine, antidepressants, benzodiazepines, antipsychotics, and sleep inductors. None of the patients were on wakefulness-promoting agents (e.g., methylphenidate).

PD was clinically divided based on the dominant feature at presentation into postural instability and gait difficulty (PIGD) and tremor-dominant subtypes [9]. The H&Y stage was divided in three groups, mild (stages 1 and 2), moderate (stage 3), and advanced (stages 4 and 5) disease [10]. LED was calculated using the following formula [11]: regular LD dose + LD CR dose × 0.75 + LD × 0.33 if entacapone + pramipexole dose × 100 + ropinirole dose × 20 + rotigotine dose × 30 + pergolide dose × 1 + bromocriptine dose × 10 + selegiline dose × 10 + rasagiline dose × 100 + amantadine dose × 1. For our research purposes, polytherapy was defined as those subjects who were receiving a combination of LD and DA. Any class of antidepressants, benzodiazepines, and antipsychotics were included. Medications considered as sleep inductors in our study were zolpidem, melatonin, or melatonin agonists.

### 2.4. Polysomnography Technique

All subjects underwent a standardized overnight, single night PSG at the NINN-Sleep Clinic after taking their normal schedule of dopaminergic treatment using a Grass Technologies Twin (version 4.5.0.27) Polysomnographer. Conventional electroencephalography electrodes (F4-M1, C4-M1, O2-M1) were placed in most patients; however, when RBD was clinically suspected, the international 10–20 system for electrode placement was applied in order to rule out epilepsy. Electrocardiography, chin, upper and lower extremities electromyography, electrooculography, pulse oximetry, and abdominal and chest respiratory effort acquisition were also registered according to the recommended specifications of the American Academy of Sleep Medicine Manual for the Scoring of Sleep and Associated Events. PSG was later scored and analyzed by a sleep medicine specialist. The diagnosis of any of the above sleep disorders was made according to the International Classification of Sleep Disorders 2nd edition [12].

For the study purposes, subjects were classified into two groups according to the presence or absence of the following sleep disorders, obstructive sleep apnea (OSA), periodic limb movement disorder (PLMD), rapid eye movements (REM), behavioral sleep disorder (RBD), or awakenings/arousals (A/A). Sleep architecture variables were also recorded, such as sleep efficiency, sleep latency, REM latency, periodic limb movement index (PLMI), Apnea-hypopnea index (AHI), sleep stages, and total sleep time. Sleep stages were divided according to the previous classification [13].

### 2.5. Statistical Analyses

For the analysis of the association between the presence or absence of any sleep disorder and sleep architecture variables (dependent variables) with the clinical and demographic variables (independent variables), a univariate analysis was used followed by a multiple logistic regression analysis. The categorical dependent variables were the sleep disorders: OSA, PLMD, RBD, and A/A. The continuous dependent variables were the sleep architecture variables: sleep efficiency, sleep latency, REM latency, PLMI, AHI, sleep stages, and total sleep time. The independent variables analyzed were the clinical and demographic data collected. The univariate analysis was performed using Student's *t*-test and a Pearson correlation. For the regression models, the independent variables were selected using the clinical plausibility and bidirectional selection criteria (forward and backward selection).

To identify predictive variables independently associated with the dichotomous sleep dependent variables we used a multiple binary logistic regression analysis. In this analysis, continuous independent variables were categorized to be included in the analysis. The Nagelkerke *r* ^2^ coefficients of the final binary regression model that offered the best prediction of the presence of each sleep disorder were calculated. The magnitude of association between the dependent and the independent variables was measured by the odds ratio (OR) and respective 95% confidence interval (CI). A *p* level < 0.05 was considered significant, but variables with levels < 0.10 were considered relevant in the final model if they showed clinical plausibility.

To identify predictive variables independently associated with the continuous sleep dependent variables we used a multiple linear regression analysis. In this analysis categorical variables were included in the model classified as 0 or 1 (for dichotomous) and 0, 1, or 2 for those showing three categories. The “*r*” and “*r* ^2^” coefficients of the final linear regression models that offered the best prediction of the variability of sleep variables were calculated. The magnitude of independent association between the sleep dependent variables and the independent variables was measured by the *B* coefficient and respective 95% confidence interval (CI).

A *p* level < 0.05 was considered significant, but variables with levels < 0.10 were considered relevant in the final model if they showed clinical plausibility. Statistical analyses were performed using commercially available statistical software (SPSS, version 20.0; SPSS, Inc., Chicago, Illinois).

## 3. Results

### 3.1. Demographic and Clinical Characteristics

Demographics, clinical characteristics, and sleep variables are shown in Table 1. Among the 55 patients analyzed, 61.8% were males with a mean age of 61.9 (SD = 10.9) years and a mean BMI of 28 (SD = 3.9). The mean disease duration was of 6.5 (SD = 6.7) years, 76.4% had a tremor-dominant PD subtype, 76.4% had mild, 18.2% had moderate, and 5.4% had severe H&Y stage. The mean MDS-UPDRS scores were Part I of 13.9 (SD = 6.1), Part II of 14 (SD = 8.5), Part III of 27.4 (SD = 14.8), Part IV of 2.4 (SD = 3.4), and a total score of 57.8 (SD = 25.4). Forty-seven percent had presence of motor fluctuations or LID. Dopaminergic therapy was carefully documented as follows: 76.4% were receiving levodopa therapy, 81.8% were on a dopamine agonist, and 60% were on polytherapy. The mean LED in our cohort was of 635.4 mg (SD = 428.6). Other medications were also documented: 10.9% were on MAO-B inhibitors, 16.4% on amantadine, 30.9% on antidepressants, 18.2% on benzodiazepines, 1.8% on antipsychotics, and 14.5% on sleep inductors.

### 3.2. Prevalence of Sleep Disorders

We observed that 56.6% of the patients had OSA, 49.1% RBD, 24.5% PLMD, and 23.6% A/A. Combinations of any of these sleep disorders were observed in 41.6% of the patients. Additionally, 3 subjects had periods of oxygen desaturation, 3 had ventricular extrasystoles, and one had RLS.

### 3.3. Predictors of Sleep-Related Variables of PD Patients

The final models of multiple binary regressions analysis showing the variables independently associated with OSA, PLMD, and RBD are shown in Table 2. There was a significant association between male gender (adjusted OR 4.4, CI 95% 0.2 to 15.8, *p* = 0.02) and lower disease duration (<5 years) (adjusted OR 4.4, CI 95% 1.1 to 17.4, *p* = 0.04) and the presence of OSA. Lower LED (<600 mg) was more associated with PLMD than higher LED (adjusted OR 6.7, CI 95% 1.5 to 30.4, *p* = 0.04). RBD was more common among patients with MDS-UPDRS Part III scores ≥ 25 (adjusted OR 4.8, CI 95% 1.2 to 18.9, *p* = 0.02) and those using LED ≥ 1000 mg (adjusted OR 11.9, CI 95% 1.7 to 81.9, *p* = 0.001). There was a nonsignificant trend (*p* = 0.06) for association between RBD and age older than 60 (adjusted OR 3.8, CI 95% 0.9 to 15.3).

The multiple linear logistic regression models showing the independent association among demographic and clinical variables and the sleep parameters of PD patients are shown in Table 3. There was a negative association among amantadine use with older age and sleep efficiency. Taken together, these two variables explain 17% of sleep efficiency of the PD patients (*p* = 0.008). The percentage of sleep stage 1 was negatively associated with dopamine agonist usage and positively associated with severe H&Y stage. These two variables explain 21% of the variation of the percentage of sleep stage 1. The percentage of sleep stage 2 showed a negative association with the MAO-B inhibitors use and MDS-UPDRS III scores. Taken together these two variables explain 14% of the percentage of sleep stage 2 variation among PD patients. The age negatively associated and explained 7% of the percentage of REM stage of our patients. The total sleep time was positively associated with male gender and negatively associated with H&Y severity. Taken together, these two variables explain 9% of the total sleep variation. The final linear regression models showed a fair association (*r* coefficient between 0.30 and 0.49) with the analyzed sleep parameters.

## 4. Discussion

A cross-sectional study of 55 PD patients was conducted at the NINN-Movement Disorders Clinic revealing a poor to fair association between clinical characteristics with sleep disorders and sleep architecture, with models explaining only 7% to 21% of the variation of the clinical variables. This study was greatly strengthened by the use of PSG. Limitations should be considered before interpreting our results. It was a cross-sectional study of Mexican patients, where methodological and ethnical biases should be contemplated, and not all described sleep disorders were studied. Nonmotor features of PD as well as social and environmental features, which could have contributed to sleep quality, were not analyzed, and also a relatively low number of patients were analyzed in our cohort. Future studies should include a larger cohort with standardized sleepiness screening scales and PSG.

### 4.1. Sleep Disorders in PD

Our findings are consistent with previous reported frequencies of sleep disorders in PD [14]. RBD is widely known to be associated with the development of Lewy body pathology and longitudinal studies have shown that eventually patients will develop signs of parkinsonism [15]. We observed that increased age, higher MDS-UPDRS part III, and higher LED were associated with RBD, consistent with previously reported findings [16]. Being male and having a shorter disease duration were associated with OSA. This is consistent with a previous report [17] but differed from other studies reporting no clinical correlations [18]. BMI did not correlate with OSA in our cohort. Variations in results may be explained by the different types of populations studied and the methodological variations applied. Other clinical factors, which could predispose to OSA (e.g., neck circumference, hip-to-waist ratio, or Mallampati score) were not analyzed and should be considered before interpreting our results. We also found that lower LED was associated with PLMD. It has been suggested that striatal dopaminergic nerve cell loss is involved in the increased number of PLMS in PD patients [19]. A previous study reported severity of the disease as clinically associated with PLMD [20] which contradicts our results, since it is common that the LED will increase as disease advances. However, there is a general consensus that the Hispanic populations overall require fewer dosages of medications and the reasons for this are unclear but could be differences in practice. Except for RBD, which is pathologically known to be a predictor of parkinsonism, the poor clinical associations we observed for the sleep disorders in our cohort suggest other factors not considered in the analysis might be better predictors or have stronger associations with these disorders in PD patients. So far, mixed findings regarding sleep disorders and their clinical associations somewhat limit the conclusions.

### 4.2. Sleep Architecture in PD

The term sleep architecture describes the structure and organization of sleep. Previous reports have shown inconsistent results regarding sleep architecture patterns in PD patients when compared to controls [14]. While some have described changes in sleep efficacy, total sleep time, sleep latency, sleep stages, sleep fragmentation, and/or frequent arousals [5], others have not shown a significant change [21]. Our results are consistent with the literature citing changes in sleep patterns of PD patients. We observed a decrease in total sleep time, in sleep efficiency, and in REM sleep stage. We also observed a prolongation in sleep latency, which can explain the prolongation in REM latency and in sleep stages 1 and 2. We also observed values indicating a moderate sleep apnea and higher PLMI. Recent studies using subjective sleep measurements have found associations between clinical characteristics and changes in sleep patterns, where the disease duration and severity of disease were important clinical factors [22]. Insomnia evaluated by the Stavanger sleepiness questionnaire was associated with longer disease duration, female gender, and depression [23]; worse Parkinson's Disease Sleep Scale (PDSS) scores were associated with higher H&Y stages [24]; and excessive daytime sleepiness using the PDSS was also related to longer disease duration [25]. Studies using objective PSG measurements have described an association between higher LED with less REM sleep stage [26] and reduced total sleep time; also age and increased H&Y stage were associated with reduced sleep efficiency [5]. Our results revealed significant associations between age with reduced sleep efficiency and reduced REM sleep stage; male gender, severity of H&Y stage, MDS-UPDRS part III scores, and medications such as dopamine agonists or MAO-B inhibitors associated with reduced sleep stages 1 and 2 and total sleep time. MAO-B inhibitors were also associated with changes in sleep stage 3. Age, gender, and indicators of disease duration were found to be associated with changes in certain variables of sleep architecture as previously described; however we did not observe associations related to the LED. Methodological differences and ethnical backgrounds could explain differences, but the reasons underlying the changes are unknown.

Sleep disorders are commonly reported in PD patients and it has been documented that variations in sleep patterns are multifactorial, suggesting that effects from increasing age, nonmotor symptoms such as depression, and dopaminergic medication may affect the mechanisms of sleep disturbances [16, 27, 28]. In what way MAO inhibitors contribute to sleep architecture is uncertain; however they may indirectly affect sleep patterns by improving depressive symptoms through its adrenocortical axis activity [29]. Our regression models revealed a poor to fair association between variables with the models and this explained only 10% to 30% of the variation. This finding was consistent with previous studies suggesting that other factors not analyzed in our cohort related to changes linked to the disease process itself were impacting sleep quality in PD patients. The exact reasons for this remain to be worked out.

### 4.3. Monoaminergic Pathway Disruption

**Figure 1:**
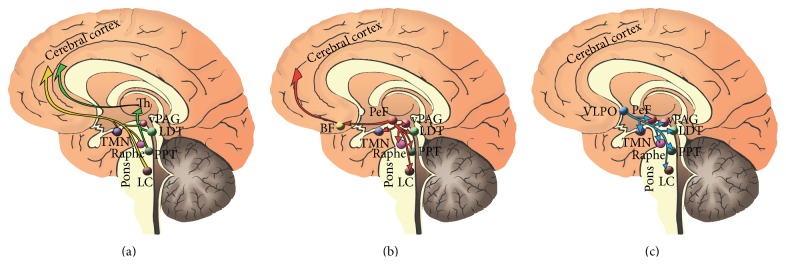
Sleep circuitry drawings of the arousal system affected in PD. Sleep arousal circuit. (a) The monoaminergic arousal system (yellow) includes neurons projecting from the noradrenergic locus coeruleus (LC), the serotoninergic dorsal raphe, the histaminergic tuberomammillary nucleus (TMN), and the dopaminergic ventral periaqueductal gray matter (vPAG). The cholinergic pedunculopontine nucleus (PPT) and the lateral-dorsal tegmental nuclei (LDT) send projections to the thalamus and promote the sensory information (green) to the cortex. (b) These ascending projections will contact the prefornical (PeF) orexin hypothalamic neurons (red) and the cholinergic basal forebrain neurons (BF), before directly innervating the cerebral cortex. Sleep inhibitory circuit. (c) The VLPO send inhibitory projections (light blue) to the components of the arousal circuit inhibiting them during sleep.

A recent study reported a significantly higher burden of alpha-synuclein in brainstem regions (locus coeruleus and raphe nuclei), hypothalamic regions (paramammillary nuclei and the posterior hypothalamus), subcortical/limbic regions (amygdala and thalamus), and also Tau pathology present in cortical regions (entorhinal cortex) of PD patients with sleep disorders when compared to patients without sleep disorders [30]. The weak clinical associations observed in our cohort confirm what previous studies have shown, where sleep quality was independent of motor functions [31]. Additionally, pathological findings observed in brain regions which disturb areas playing roles in arousal and wakefulness suggest that different brain circuits or degenerative brain processes are affecting sleep physiology in PD other than dopaminergic circuitry. Numerous wide projections of neurons release different types of neurotransmitters and neuropeptides to regulate this intricate phenomenon of sleep in the brain. Interestingly, the pathologically alpha-synuclein affected brain regions in PD patients with sleep disorders have been shown in some studies to be consistent with the ascending arousal system monoaminergic pathways, including the noradrenergic locus coeruleus, serotoninergic raphe nuclei, dopaminergic periaqueductal gray matter, and histaminergic tuberomamillary nucleus [32]. Neurons on these sites send projections to basal forebrain and cerebral cortex through the lateral hypothalamus, which is also affected by alpha-synuclein. Overall, these observations suggest that monoaminergic pathways likely have a significant impact on PD sleep related disorders.  Figure 1 theoretically represents the possible affected sleep pathways in PD.

In summary, sleep disorders and abnormal sleep architecture are common findings in PD patients despite their clinical characteristics. Other suggested circuits besides the dopaminergic pathways can impact the quality of sleep. It is critical to better understand the complex pathophysiology of sleep in PD patients in order to develop effective treatment strategies to improve a patient and a caregiver's quality of life.

## Figures and Tables

**Table 1:** Demographic, clinical, and sleep variables of our PD cohort.

Variables	Mean (±SD)
Age	61.9 (10.9)
BMI	28 (3.9)
Disease duration	6.5 (6.7)
UPDRS	
Part I	13.9 (6.1)
Part II	14 (8.5)
Part III	27.4 (18.4)
Part IV	2.3 (3.4)
LED, mg	635.4 (428.6)

Sleep-related variables^a^	Mean ± SD
	(minimum–maximum)

Sleep latency, min	29.7 ± 39.6 (1–221.5)
REM latency, min	187.3 ± 114.3 (0–468.5)
Sleep efficiency, %	68.1 ± 18.6 (6–94.3)
Total sleep time, min	345.4 ± 109.4 (6–94.3)
Stage 1, %	11.9 ± 9.7 (0–47)
Stage 2, %	61.2 ± 16.9 (16.8–100)
Stage 3, %	13.4 ± 10.2 (0–46.4)
REM, %	13.4 ± 7.6 (0–28.2)
AHI	19.9 ± 21.2 (0–75.4)
PLMI	39.4 ± 105.6 (0–540)

	*n* = 55 (%)

Sleep disorders^b^	
OSA	31 (56.6)
RBD	27 (49.1)
PLMD	13 (24.5)
Awakening/arousals	13 (23.6)
Male	34 (61.8)
Tremor-dominant PD subtype	42 (76.4)
H&Y stage	
Mild	42 (76.4)
Moderate	19 (18.2)
Severe	03 (5.4)
Motor fluctuation of LID	26 (47)
Dopaminergic treatment	
Levodopa therapy	42 (76.4)
Dopamine agonists	45 (81.8)
Polytherapy	33 (60)
Other medications	
Antidepressants	17 (30.9)
Benzodiazepines	10 (18.2)
Amantadine	9 (16.4)
Sleep inductors	8 (14.5)
MAO-B inhibitors	6 (10.9)
Antipsychotics	1 (1.8)

^a^REM: rapid eye movement; AHI: apnea-hypopnea index; PLMI: periodic limb movement index.

^b^Twenty-three patients (41.6%) had more than one sleep disorder. Three patients had oxygen desaturation, three had ventricular extra systoles, and one had restless leg syndrome. OSA = obstructive sleep apnea; RBD = REM behavioral sleep disorder; PLMD = periodic limb movement disorder.

**Table 2:** Multiple binary logistic regression models showing the independent association among demographic and clinical variables and sleep disorders of PD patients.

Variable	Adjusted OR	“*p*” value
	(CI 95%)	
OSA^a^		
Male	4.4 (1.2–15.8)	0.02
Disease duration < 5 years	4.4 (1.1–17.4)	0.04

	Nagelkerke *r* ^2^ = 0.30	

PLMD^b^		
LED < 600 mg	6.7 (1.5–30.4)	0.02

	Nagelkerke *r* ^2^ = 0.20	

RBD^c^		
≥60 years of age	3.8 (0.9–15.3)	0.06
MDS-UPDRS part III score ≥ 25	4.8 (1.2–18.9)	0.02
LED ≥ 1000 mg	11.9 (1.7–81.9)	0.01

	Nagelkerke *r* ^2^ = 0.30	

^a^OSA: obstructive sleep apnea; ^b^PLMD: periodic limb movement disorder; ^c^RBD: REM behavioral sleep disorder.

**Table 3:** Multiple linear logistic regression models showing the independent association among demographic and clinical variables and the sleep parameters of PD patients.

Variable	*B* coefficients	95% CI	“*p*” value
Sleep efficiency			
Constant	105.17 (13.57)		<0.0001
Amantadine	−11.37 (6.29)	−24.0 to 1.26	0.07
Age, years	−0.57 (0.22)	−1.0 to −0.14	0.01

		**r = 0.41**; **adjusted** **r** ^2^ = 0.17	**0.008**

Stage 1%			
Constant	18.49 (2.73)	13.03 to 23.96	<0.0001
Dopamine agonist	−9.08 (3.03)	−15.17 to −2.99	0.004
Severe H&Y stage^a^	15.90 (5.13)	5.57 to 26.24	0.003

		**r = 0.49**; **adjusted** **r** ^2^ = 0.21	**0.01**

Stage 2%			
Constant	67.22 (2.82)	61.55 to 72.88	<0.0001
MAO-B inhibitors^b^	−16.25 (6.85)	−29.98 to −2.51	0.02
MDS-UPDRS III^c^	−11.67 (0.44)	−20.47 to −2.66	0.01

		**r = 0.42**; **adjusted** **r** ^2^ = 0.14	**0.007**

Stage 3%			
Constant	10.77 (1.77)	7.23 to 14.32	<0.0001
MAO-B inhibitors^b^	8.75 (4.29)	0.14 to 17.35	0.04
MDS-UPDRS III^c^	4.69 (2.78)	−0.89 to 10.27	0.09

		**r = 0.33**; **adjusted** **r** ^2^ = 0.07	**0.05**

REM stage %			
Constant	26.2 (5.73)	14.69 to 37.68	<0.0001
Age	−0.21 (0.09)	−0.39 to −0.02	0.02

		**r = 0.30**; **adjusted** **r** ^2^ = 0.07	**0.03**

Total sleep time, minutes			
Constant	329.05 (18.26)	292.42 to 365.69	<0.0001
Male	58.59 (28.95)	0.48 to −116.70	0.05
Severe H&Y stage^a^	−109.58 (61.95)	−233.90 to −14.73	0.08

		**r = 0.35**; **adjusted** **r** ^2^ = 0.09	**0.03**

^a^H&Y = Hoehn & Yahr stage. Moderate and severe affected patients were grouped as severe H&Y stage; ^b^MAO-B = monoamine oxidase type B; ^c^MDS-UPDRS = Movement Disorders Society-Unified Parkinson's Disease Rating Scale, Part III.
